# Skin rash by gefitinib is a sign of favorable outcomes for patients of advanced lung adenocarcinoma in Japanese patients

**DOI:** 10.1186/2193-1801-2-22

**Published:** 2013-01-23

**Authors:** Yasoo Sugiura, Etsuo Nemoto, Osamu Kawai, Yasuyuki Ohkubo, Hisae Fusegawa, Shizuka Kaseda

**Affiliations:** 1 Pulmonary and Thoracic Surgery, National Hospital Organization, Kanagawa National Hospital, 666-1 Ochiai, Hadano, Kanagawa, 257-8585 Japan; 2 Respiratory Medicine, National Hospital Organization, Kanagawa National Hospital, 666-1 Ochiai, Hadano, Kanagawa, 257-8585 Japan

**Keywords:** Gefitinib, Skin rash, Lung cancer, Adenocarcinoma, Response rate, Predictive factor

## Abstract

Skin rash is one of the notorious adverse events of gefitinib as well as other epidermal growth factor receptor tyrosine kinase inhibitors. The differences of response rate and frequency of adverse events between ethnic groups are well known. Some reports demonstrated the correlation between development of rash and efficacy in Caucasian patients treated with erlotinib, gefitinib or cetuximab. We analyzed clinical course of Japanese patients of lung adenocarcinoma in order to assess the relation between adverse events and efficacy of gefitinib. Between January 2008 and June 2012, 24 Japanese patients administered gefitinib 250 mg daily. The adverse events were evaluated in accordance with Common Terminology Criteria For Adverse Events v4.0 (CTCAE). Objective response to gefitinib was evaluated with using computed tomography every 1–2 months. The relationship between each adverse event and objective response was examined by chi-square test. The Log-rank Test was used to assess the relationship between the presence of skin rash and overall survival. Twenty four patients with a median age of 67 years (range 55–89) entered were 16 female and 8 male patients; the pathological diagnosis of all patients was adenocarcinoma. Skin rash in CTCAE occurred in 10. The objective response and overall survival among the patients with skin rash was significantly superior to the patients without skin rash. Skin rash by gefitinib correlates with improved clinical outcomes among advanced lung adenocarcinoma patients.

## Introduction

Gefitinib has been already the first line treatment as one of the epidermal growth factor receptor (EGFR) tyrosine kinase inhibitors (TKI) for lung adenocarcinoma cancer with EGFR mutation (Keedy et al. 2011). EGFR mutation was found to be statistically significantly more frequent in patients of East Asian origin versus other ethnicities (30 versus 8%) (Shigematsu et al. 2005). Therefore, the percentage of responders to EGFR-TKI almost paralleled the percentage of patients with EGFR mutations (Jiang 2009; Kris et al. 2003, Sequist et al. 2008; Tamura et al. 2008). On the other hand, interstitial lung disease (ILD) has been reported in Japanese NSCLC patients receiving gefitinib at higher rates than outside Japan (Ando et al. 2006; Kudoh et al. 2008; Park and Goto 2006). Because ILD induced by gefitinib is high mortality, the ethnic differences of ILD induced by gefitinib is reported well. Although the data of Fukuoka et al. have shown that the adverse event profile for gefitinib in Asia and non-Asia patients is similar (Fukuoka et al. 2003), the relationship between the efficacy and adverse events of gefitinib in the respect of the ethnic differences are still unclear.

EGFR-TKI causes different adverse events from other anti-cancer agents, though molecular targeted anti-cancer drug was expected to be less harm than the other cytotoxic anti-cancer drugs (Forsythe and Faulkner 2004). Indeed, cytotoxic adverse events such as myelosuppression occur in lower rate (Kim et al. 2008; Thatcher et al. 2005). However, skin trouble such as skin rash, desquamation and itching, and digestive symptoms such as diarrhea, nausea and liver enzyme elevation emerge in higher rate (Forsythe and Faulkner 2004; Van Zandwijk 2003). Skin rash is notorious as an adverse event of EGFR-TKI and is noted in up to two-thirds of patients receiving any of these agents although severe in only 5 to 10% (Jacot et al. 2004). Gefitinib, erlotinib and cetuximab correlates between development of rash and efficacy (Argiris and Mittal 2004; Cadranel et al. 2009; Chiu et al. 2005; Janne et al. 2004; Stintzing et al. 2012; Wacker et al. 2007; West et al. 2006). The most patients of these studies were Caucasian, and there are very few reports treating with Asian patients. Herein, we retrospectively assessed the relation between each adverse event and efficacy of gefitinib.

## Patients and methods

### Patients

Present study was a retrospective analysis of all patients with pathologically proven adenocarcinoma at the Kanagawa National Hospital from January 2008 to June 2012. All patients were Japanese whose ethnic origins were Asian. Gefitinib was administered at a dose of 250 mg daily to 24 patients with lung adenocarcinoma; 10 with post-operative recurrence and 14 inoperative advanced cases. There were 16 women and 8 men with ages ranging from 55 to 89 years (median: 67 years old) (Table 1). Informed consents of the patients were obtained in accordance with institutional guidance based on the Helsinki Declaration on admission. The relevant institutional review board had approved this study.

**Table 1:** **Clinical characteristics**

	Total
Sex	
Female	16
Male	8
Age (median)	55-89 (67)
Ethnic origin (cases)	
Asian	24
Histology (cases)	
Adenocarcinoma	24
EGFR mutation (cases)	
Exon 18 G719X	1
Exon 19 deletion	8
Exon 21 L858R	7
None	3
Unknown	5
Operative cases and inoperative cases	
Post-operative recurrent cases	10
Inoperative advanced cases	14
Performance Status (cases)	
0	15
1	6
2	1
3	2
4	0
Metastasis or recurrence site (cases)^*^	
Bone	8
Pleurisy	6
Lung	6
Lymph node	6
Brain	6
Adrenal	2
Liver	1
Only tumor marker elevation	2

* There are some overlappings EGFR: Epidermal Growth Factor Receptor.

## Methods

Follow-up assessments included physical examination, complete blood counts, blood chemistry every 2–4 weeks and tumor assessment by computed tomography were done every 1–2 months.

Tumor response was assessed as complete response (CR), partial response (PR), stable disease ≧12 weeks (SD) or progressive disease (PD) in accordance with the standard Response Evaluation Criteria In Solid Tumours (RECIST). Adverse events were evaluated and graded using Common Terminology Criteria For Adverse Events v4.0 (CTCAE). Skin rash and the grade were defined as following criteria according to the CTCAE: grade 1, macular or popular eruption or erythema without associated symptoms; grade 2, macular or popular eruption or erythema with pruritus or other associated symptoms covering ≤50% of body surface; grade 3, symptomatic generalized erythroderma or macular, popular or vesicular eruption or desquamation covering >50% of body surface; grade 4, generalized exfoliative dermatitis or ulcerative dermatitis. After grade 3 adverse event emerged, the dose of gefitinib was reduced to 250 mg on alternate days or administration was discontinued.

Overall survival (OS) was evaluated for the period from diagnosis as inoperative advanced lung cancer or post-operatively recurrent lung cancer to the date of death, referring to Dictionary Cancer Terms of the National Cancer Institute. Kaplan-Meier survival curves were drawn for OS, compared by means of Log-rank test. For comparisons of proportions, chi-square test and Fisher's exact test were used. All results were considered significant at *p-*values of less than 0.05. All statistical analysis was performed using the Stat Mate IV software program version 4.01 (ATM, Inc, Bunkyo, Tokyo, Japan).

## Results

### Patient characteristics

From January 2008 to June 2012, gefitinib was administered to 24 patients with lung adenocarcinoma; 10 with post-operative recurrence and 14 inoperative advanced cases. There were 16 women and 8 men with ages ranging from 55 to 89 years (median: 67 years old). The ethnic origin of all patients was Asian (Table 1).

The detail mutations of EGFR was that one case had exon 18 G719X, 8 had in exon19 deletion, 7 had in exon 21 L858R, 3 did not have any EGFR mutations and 5 were not examined. In ECOG PS, 21cases were in PS 0–1 and 3 were in PS 2–3.

Cases with previous chemotherapy were 10 and chemotherapy naïve cases were 14. The regimen of the previous chemotherapy was that 7 cases were administered platinum anti-cancer agent and the third generation anti-cancer agent, a case were administered gemcitabine (GEM) and vinorelbin (VNR), 2 cases were given tegafur-uracil (UFT) as the first-line treatment, a case was administered pemetrexed (PEM) and a case was administered docetaxel (DTX) as the second-line treatment.

### Adverse events

In all 24 cases, skin trouble emerged in 10 cases (41.7%), liver damage occurred in 9 cases (37.5%), diarrhea emerged in 4 cases (16.7%) and acute lung injury emerged in 2 cases (8.3%) (Table 2). The grade of each case with skin rash was less than 2. Since the liver damage was grade 3 in all 9 cases in CTAE, the dose was reduced to the alternate days. The grade of each case with diarrhea was also less than 2. Lung injury emerged in 2 cases was grade 4 and they died.

**Table 2:** **Adverse events after administration of gefinitib**

(cases)	Total (%)
Skin rash	10 (41.7)
Liver damage	9 (37.5)
Diarrhea	4 (16.7)
Acute lung injury	2 (8.3)

### Response to treatment and overall survival

**Figure 1:**
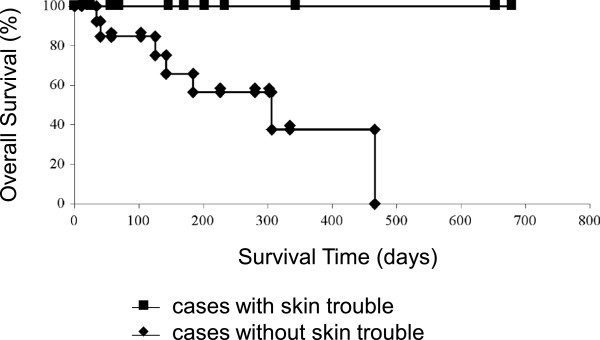
**Overall survival of advanced inoperative or postoperative recurrent lung adenocarcinoma patients with skin rash and without skin rash.**

Two patients achieved CR and 11 did PR. Four patients resulted in SD and 7 did in PD as their best response. The objective response rates (ORR) of all cases, cases with skin rash and without skin rash were 54.2%, 100% and 21.4% respectively (Table 3). Overall survival of the cases with skin rash was significantly superior to the cases without skin rash (*p* = 0.012, Figure 1).

**Table 3:** **Objective response rate in cases administered gefitinib 250 mg/day and dose redused due to liver damage**

	Skin rash		
	+	-	Total
RECIST			
CR	2	0	2
PR	8	3	11
SD	0	4	4
PD	0	7	7
CR + PR (%)	10 (100)	3 (21.4)	13 (54.2)

*RECIST* Response Evaluation Criteria in Solid Tumors, *CR* Complete Response, *PR* Partial Response, *SD* Stable Disease, *PD* Progressive Disease.

## Discussion

The response rate and adverse events have the ethnic differences in gefitinib for advanced lung adenocarcinoma. For instance, the incidence of the EGFR mutation is higher frequent in the Asian patients than the other origin patients and ILD induced by gefitinib occurs more frequently in Japan more than outside Japan (Ando et al. 2006; Jiang 2009; Kris et al. 2003; Kudoh et al. 2008; Park and Goto 2006; Sequist et al. 2008; Shigematsu et al. 2005; Tamura et al. 2008). On the other hand, the correlation between development of rash and efficacy in patients treated with erlotinib or cetuximab was demonstrated in some reports (Stintzing et al. 2012; Wacker et al. 2007). Dudek reported that skin rash and bronchioalveolar histology correlates with clinical benefit in patients with gefitinib as a therapy for previously treated advanced or metastatic non-small cell lung cancer (Dudek et al. 2006). However, the ethic population of these reports was Caucasian and nobody knows whether it is applicable for Japanese patients. We analyzed clinical courses of Japanese patients of lung adenocarcinoma in order to assess the relation between each adverse event and efficacy of gefitinib, because gefitinib is one of the first-line treatments for advanced non-small cell lung cancer with EGFR mutation in guidelines of American Society of Clinical Oncology (ASCO) (Keedy et al. 2011) and the Japan Lung Cancer Society, and adenocarcinoma is the most common pathological type in lung cancer (Jemal et al. 2011).

We could indicate two significant evidences in Japanese patients that skin rash predicted a good prognosis in the patients of lung adenocarcinoma treated with gefitinib. First of all, patients with skin rash achieved significantly higher ORR than patients without skin rash in advanced inoperative or postoperative recurrent lung adenocarcinoma treated with gefitinib (*p* = 0.001458, Table 4). Other adverse events and ORR did not show relationship. In the second, after following more than 2 years, overall survival of the cases with skin rash was significantly superior to the cases without skin rash (*p* = 0.012, Figure 1). From the above, these analyses document the relationship between skin rash during gefitinib therapy and improvements in ORR and survival.

**Table 4:** **Relationship between each adverse event and objective response rate**

	Skin rash		Diarrhea		Liver damage	
	+	-	+	-	+	-
CR, PR	10	3	2	11	6	7
SD, PD	0	11	2	9	3	8
*p*-value	0.001458		0.636		0.3001	

*CR* Complete Response, *PR* Partial Response, *SD* Stable Disease, *PD* Progressive Disease.

One important question is that why the skin rash emerged on the normal skin of the patients of lung adenocarcinoma with EGFR mutation, although did not without EGFR mutation. Indeed, in this study, 3 cases without EGFR mutation treated by gefitinib resulted in PD and skin rash did not occurred at all. Francasso reported that administration of single doses (250–500 mg/m^2^) of cetuximab resulted in a dose-dependent decrease in EGFR protein expression levels in skin (Fracasso et al. 2007). Biopsy of the lesions of skin rash induced by gefitinib showed prominent keratin plugging in dilated infundibula of hair follicles, a superficial purulent folliculitis and disordered differentiation with focal parakeratosis were seen (Albanell et al. 2002, Van Doorn et al. 2002). These reports suggested that EGFR-TKI interrupted the function of EGFR in not only tumor, but also on normal skin, and consequently skin rash emerged.

In clinical practice of treating advanced lung cancer, specimen of the tumor by transbronchial lung biopsy or cell block of pleural effusion by thoracocentesis are occasionally insufficient or inappropriate to detect EGFR mutation (Aisner et al. 2011).The efficacy of the treatment with gefitinib may be predictable in advance, if the interaction between the skin and gefitinib in lung cancer patients can be examined before initiating therapy. In other words, skin biopsy would replace transbronchial lung biopsy.

In conclusion, ORR and OS of the advanced inoperative or postoperative recurrent patients of lung adenocarcinoma with skin rash by gefitinib were significantly superior to the cases without skin rash. Further examinations are necessary in order to generalize our results and hypothesis to clinical practice, because our study population is small and our hospital is not a cancer center but a general municipal hospital.

## Conclusion

Skin rash by gefitinib for the advanced inoperative or postoperative recurrent patients of lung adenocarcinoma is a significant predictive factor of objective response and prognosis.
